# Management of a metacarpal oblique fracture in an elite cyclist

**DOI:** 10.1093/omcr/omae044

**Published:** 2024-05-20

**Authors:** Stuart L Trager, Dawn T Gulick

**Affiliations:** Department of Orthopaedics, Jefferson Health-Northeast, 380 North Oxford Valley Road, Langhorne, PA, USA; Department of Physical Therapy, One University Place, Chester, PA, USA

## Abstract

Metacarpal fractures are common in contact sports but are not often seen in elite cyclists. This case report depicts a 32-year-old elite cyclist injured when forced into a race course metal barricade. The diagnosis was an oblique fracture of the third metacarpal. This report chronicles the diagnosis, clinical signs and symptoms, and application of custom splinting to maximize athletic participation during the rehabilitation process.

## INTRODUCTION

The metacarpal (MC) bones of the hand articulate with their neighboring carpal bones to form three mobile segments (digits 1, 4, and 5) and two stiffer central pillars (digits 2 and 3) [1]. Each MC can be divided into three anatomic segments: base or proximal end, shaft or middle section, and head or distal end. Metacarpal fractures are a common hand injury in the athletic population and usually result from a direct blow or fall onto the hand [1]. A direct blow to the dorsum of the hand usually results in a transverse fracture, while axial or torsional forces may result in spiral or oblique fractures [2].

Physical presentation of an MC fracture includes pain, edema, ecchymosis, limited hand mobility, and asymmetry of the knuckles [3]. Malrotation of the fracture may cause scissoring (of the digits) which is most easily identified with active digital flexion. In more subtle cases or when swelling and pain limit the ability to flex the fingers, malrotation may be identified by alterations in the plane of the nails when viewed form the end of the finger [3]. Radiographs to identify a fracture include posteroanterior (PA), lateral, and oblique views. Given the concavity of the palmar arch, a 30° pronated lateral view for second and third metacarpal fractures and a 30° supinated lateral view for fourth and fifth metacarpal fractures are helpful [4].

## CASE REPORT

This case report involves a 32-year-old right-hand dominant, elite female cyclist. The mechanism of injury was forceful contact of the right hand into a metal barricade used to delineate the boundary of a road race course. The athlete experienced severe pain but remained in the race despite having difficulty braking, shifting gears, and weight bearing through the hand was extremely painful.

**Figure 1:**
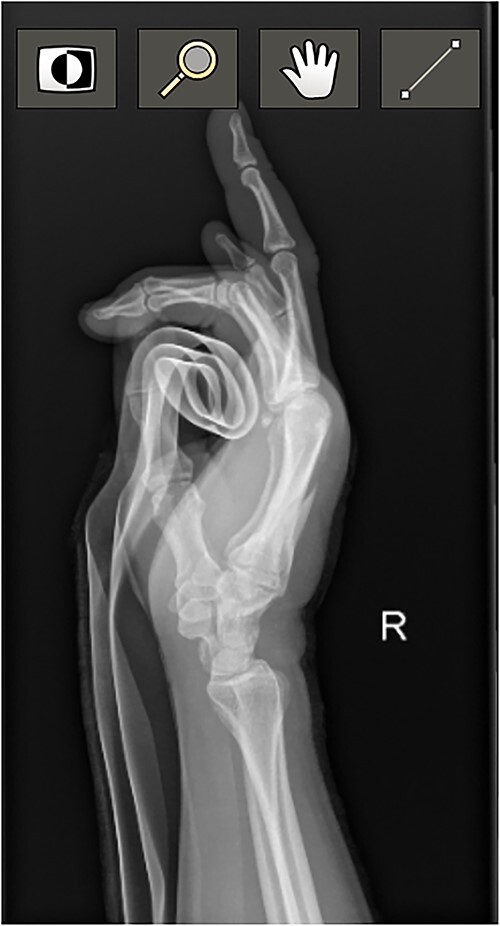
Diagnostic Radiograph 1.

**Figure 2:**
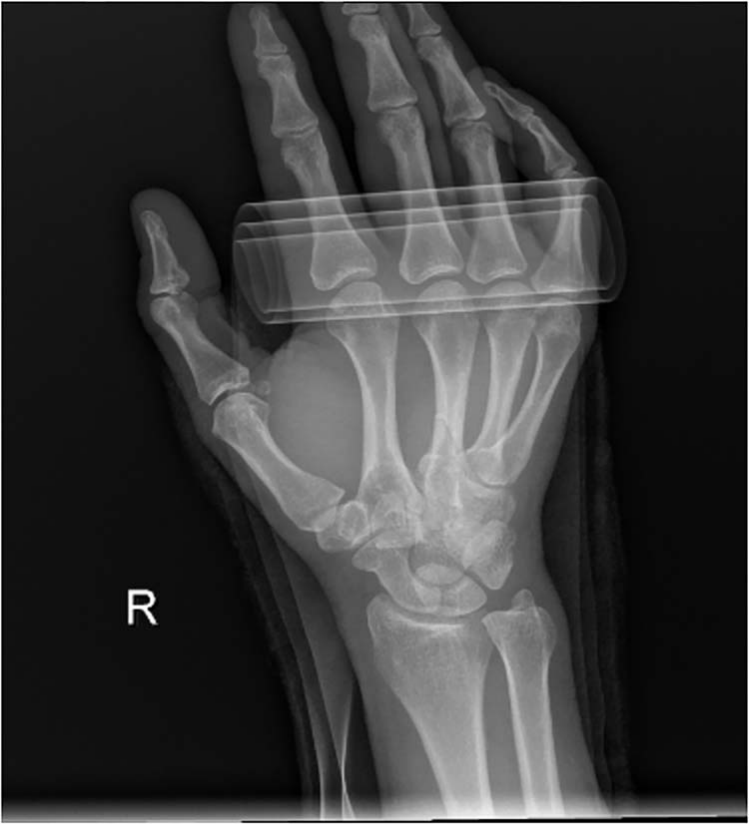
Diagnostic Radiograph 2.

**Figure 3:**
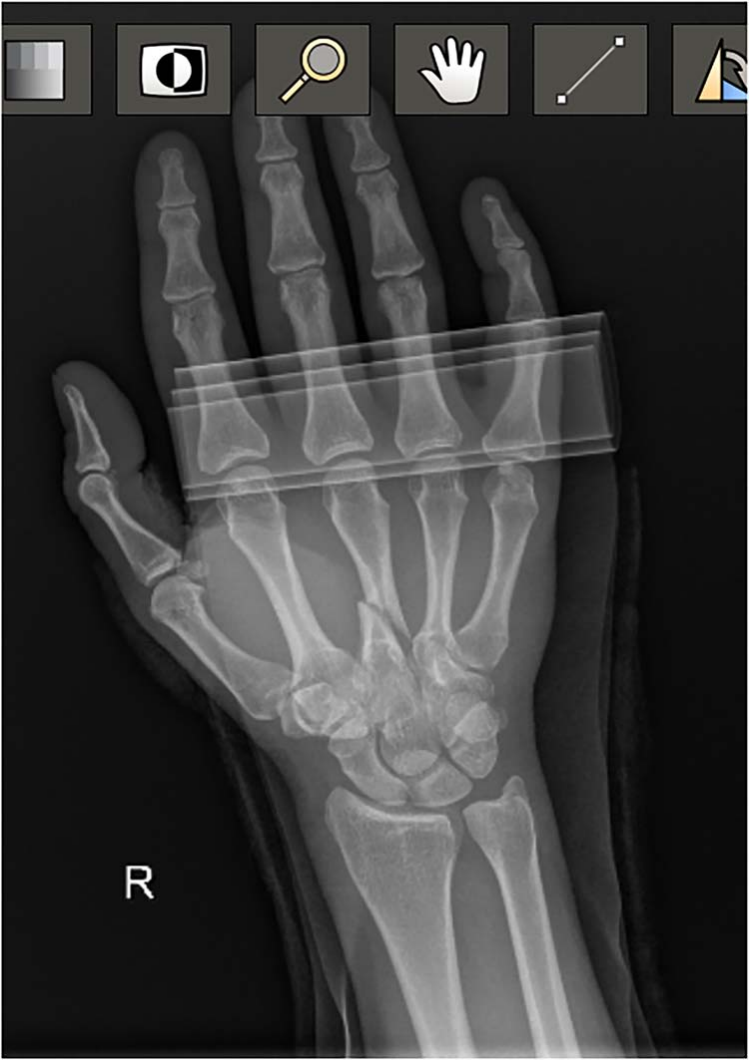
Diagnostic Radiograph 3.

**Figure 4:**
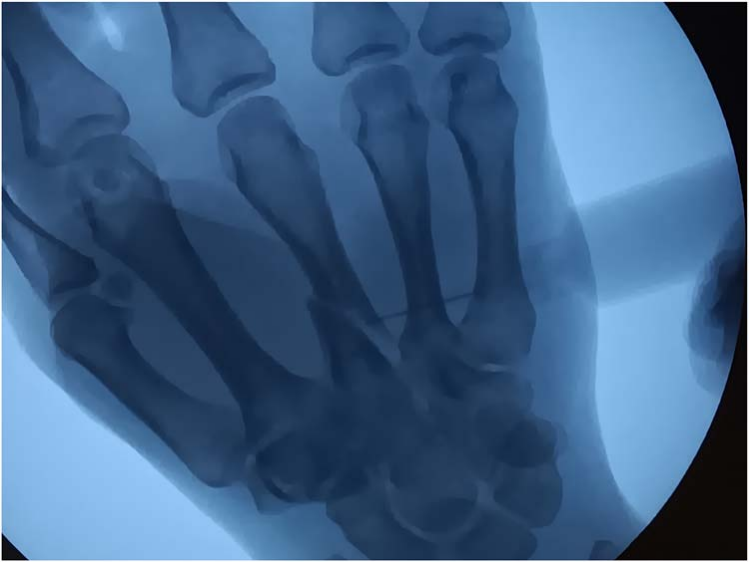
Fluoroscope Image Setting the Fracture.

At the conclusion of the race, she was evaluated by the medical director. The dorsum of the hand was extremely swollen and pain was rated as a 7 (0–10 scale) on a numeric pain rating scale. Range of motion (ROM) of her wrist and thumb were functional but her third metacarpal was tender to palpation with both active and passive motion. Sensation and circulation were intact. Ice was applied, the hand was immobilized with a SAM splint, and the athlete was referred to the emergency room. A third MC oblique fracture was identified on radiograph (Figs 1–3). After a hematoma block with lidocaine, a closed fracture reduction was performed under fluoroscopy (Fig. 4).

**Figure 5:**
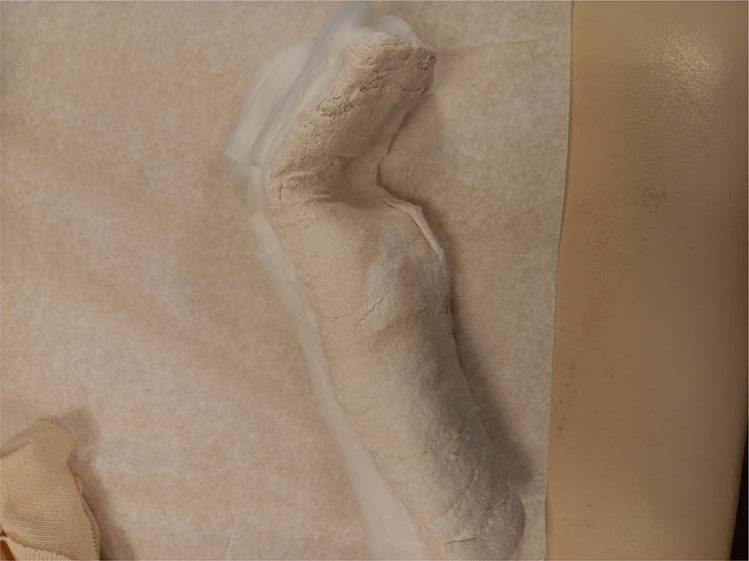
Splint Fabricated in the Emergency Department on the Day of Injury.

The athlete was then splinted in slight wrist extension, approximately 70 degrees of metacarpal-phalangeal flexion, and interphalangeal (IP) extension (Fig. 5). Digits 2 through 5 were all included in the splint. The splint was removed several times daily to apply ice for edema management.

**Figure 6:**
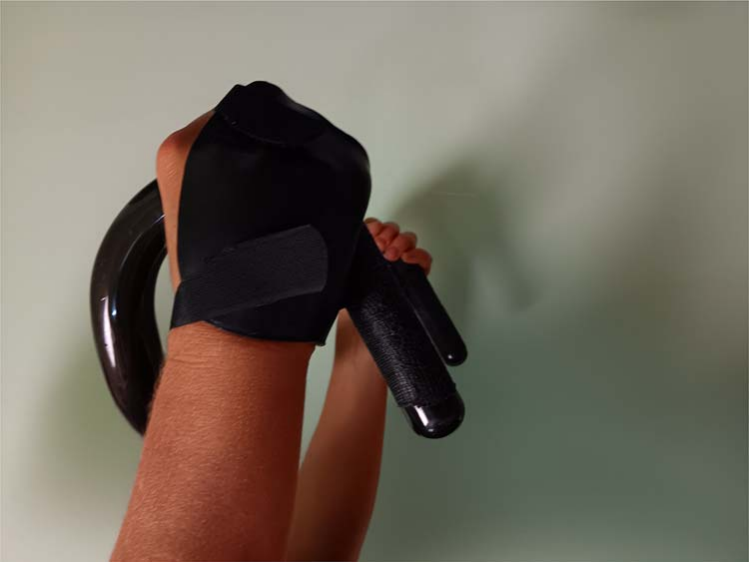
Low Profile Splint for Training and Racing.

Over the next five days, the athlete trained on a stationary bike in the splint, using aerobars to enable weightbearing on her forearms instead of her hands. Pain level was a 1 (0–10) without medication. As a result of a racing commitment (prior to fracture) to compete five days post fracture, the athlete underwent additional radiographs to confirm alignment prior to fabrication of a low-profile splint. This thermoplastic splint fabricated enabled her to grip the handlebars of a track bike (Fig. 6). Racing on a track bike with this injury was possible since a track bike utilizes a fixed gear with no brakes. The brace was effective (defined as allowing athlete improved ability to control the bike while at the same time optimizing fracture stability and pain control). As an additional precaution, the IP of digits 3 and 4 were ‘buddy taped’ together to minimize the risk of rotation with finger flexion. Although pain was minimal, swelling had to be managed at the conclusion of the event.

On day 8, the athlete began heating with gentle, active, pain-free ROM. On day 14, the athlete began tendon glides 200×/day. Until day 22, all training was performed using aerobars on a trainer. On day 23, follow-up radiographs were performed to assess healing prior to the return to road training, i.e. the need for brakes and shifting gears. The third metacarpal fracture alignment was well maintained with shortening within acceptable range (2–5 mm) [2]. On day 27, modifications to the splint were performed to accommodate improved tolerance for flexion, i.e. allow more MCP mobility for forceful braking. Therapeutic exercises were implemented to increase ROM and strength. Grip strength was assessed with a blood pressure cuff (cuff pumped up to 20 mm Hg then squeezed by the athlete; maximal pressure generated was recorded). The uninvolved hand generated 208 mm Hg, while the involved hand recorded 60 mm Hg (72% deficit). Putty and CanDo digi-flex (Fabrication Enterprises, White Plains, NY) devices were utilized 3-4x/day in conjunction with the application of heat. Pain level remained at a 0/10, however jarring due to road conditions were moderately uncomfortable.

By day 33, training on the track was performed without the splint and by day 40, road training was performed without the splint. Budding taping of 3^rd^ and 4^th^ digits continued. Grip strength was again assessed and reported to be 80 mm Hg (60% deficit). MCP and IP flexion was 90 degrees throughout. Full extension of the MCP and IPs could also be achieved with minimal residual edema of the digits. By day 42, the athlete resumed full participation without any protective splinting/taping and had no residual effects. Strengthening exercises were to be continued until hand strength and dexterity were symmetrical.

## DISCUSSION

Cyclists most commonly fracture the clavicle and scaphoid when involved in a crash. This case involves the fracture of the shaft of the third MC as a result of colliding with a metal race course barricade. This case report outlines the pathway of diagnosis, splinting, and rehabilitation used to keep the athlete progressing toward elite performance for international competition. It demonstrates the adaptions made to maintain fitness, continued participation in training and racing, while still protecting the fracture for proper healing. At the conclusion of this case report, the athlete still had some work to do to recover her strength and dexterity but she was showing consistent gains. Based on the literature, consensus on the acceptable nonoperative parameters following closed reduction of metacarpal shaft fractures of the third MC include angulation of less than or equal to 10–20 degrees and shortening of 2–5 mm [2]. With fracture alignment within these parameters, no clinical evidence of malrotation, and an aggressive rehabilitation protocol yielding full early range of motion, the athlete is expected to have an excellent outcome without significant deficits.
